# Severe MgADP Inhibition of *Bacillus subtilis* F_1_-ATPase Is Not Due to the Absence of Nucleotide Binding to the Noncatalytic Nucleotide Binding Sites

**DOI:** 10.1371/journal.pone.0107197

**Published:** 2014-09-22

**Authors:** Toru Ishikawa, Yasuyuki Kato-Yamada

**Affiliations:** 1 Department of Life Science, Rikkyo University, Toshima-ku, Tokyo, Japan; 2 Research Center for Life Science, Rikkyo University, Toshima-ku, Tokyo, Japan; Loyola University Medical Center, United States of America

## Abstract

F_1_-ATPase from *Bacillus subtilis* (BF_1_) is severely suppressed by the MgADP inhibition. Here, we have tested if this is due to the loss of nucleotide binding to the noncatalytic site that is required for the activation. Measurements with a tryptophan mutant of BF_1_ indicated that the noncatalytic sites could bind ATP normally. Furthermore, the mutant BF_1_ that cannot bind ATP to the noncatalytic sites showed much lower ATPase activity. It was concluded that the cause of strong MgADP inhibition of BF_1_ is not the weak nucleotide binding to the noncatalytic sites but the other steps required for the activation.

## Introduction

FoF_1_-ATPase/synthase (FoF_1_) catalyzes ATP synthesis from ADP and inorganic phosphate coupled with the H^+^ flow driven by the electrochemical gradient of H^+^ across cellular membranes. FoF_1_ consists of a water-soluble F_1_ part (F_1_-ATPase) connected to a membrane-embedded H^+^ channel, Fo [1]–[3]. F_1_-ATPase consists of α_3_, β_3_, γ, δ and ε subunits and its hydrolysis of one ATP molecule at a catalytic site on the β subunit drives a discrete 120° rotation of the γε subunits relative to the α_3_β_3_δ [4]. In FoF_1_, rotation of the rotor subunits of F_1_ (γ and ε) is transferred to the c subunit ring of Fo to couple ATP synthesis/hydrolysis and H^+^ flow.

The catalytic mechanism of ATP synthase has been extensively studied by structural studies and single-molecular experiments and the mechanism of the regulation of ATP synthase becomes attracting more interests. Several regulatory mechanisms are known: The mitochondrial ATP synthase has specific regulatory protein called IF_1_, which prevent ATP hydrolysis; The chloroplast ATP synthase has a pair of cystein residues in the γ subunit and the formation of the disulfide between them inhibits the activity; The ε subunit of bacterial and chloroplast ATP synthase inhibits ATP hydrolysis: and so on. Among them, the most prominent is MgADP inhibition [5]–[7]. When the ATP hydrolysis product, MgADP, is tightly bound at a catalytic site, the F_1_-ATPase is stalled. It is a common mechanism among all ATP synthases examined so far. Several factors are known to affect MgADP inhibition; Sodium azide stabilizes MgADP inhibition [6]: A detergent lauryldimethylamine *N*-oxide (LDAO) releases MgADP inhibition [8]: Incubation with Pi reduces MgADP inhibition [9]: and so on. It is also known that nucleotide binding to the noncatalytic nucleotide binding sites on the α subunits facilitate escape from MgADP inhibition [10]. Thus, in the ATP hydrolysis reaction, initial high activity decreases with time due to the MgADP inhibition. Then F_1_ reaches equilibrium between active and MgADP inhibited states, resulting in lower steady-state activity compared to the initial one [5], [11].

Our recent study revealed that the ATPase activity of F_1_-ATPase from *Bacillus subtilis* (BF_1_) is highly suppressed by the MgADP inhibition [12]. The initial ATPase activity, which is not inhibited by the MgADP inhibition, falls down rapidly to several percent in the steady state [12]. That is very large inactivation compared to other F_1_-ATPases because they only fall into half, one third or so [5], [11]. LDAO activates BF_1_ more than a hundredfold [12] and this activation is also very large compared to those of other F_1_-ATPases (only several fold) [8]. Due in part to the strong MgADP inhibition, BF_1_ has a strange ATP concentration dependency of steady-state ATPase activity, the ATPase activity at 20∼100 µM ATP is lower than those at 1∼10 µM or 200∼5000 µM [12]. Interestingly, the ε subunit does not inhibit but activates BF_1_ by releasing MgADP inhibition [12]. In bacterial ATP synthases, the relationship between these two inhibitions must be very important to gain proper regulation fit for the physiological demand. Thus, studying such a characteristic behavior of BF_1_ will help us to understand how the regulation of ATP synthase varies depending on the environment where the source organisms live.

Studies with F_1_-ATPases from other species showed that the ATP binding to the noncatalytic site promotes release of inhibitory MgADP from catalytic sites and results in the substantial activation [10], [13]. A mutant F_1_-ATPase from thermophilic *Bacillus* PS3 (TF_1_) that cannot bind nucleotide to the noncatalytic site showed large initial inactivation that reached to essentially no steady-state activity [13]. In eubacterial V-type ATPases, which is thought to have the same origin as F_1_-ATPases, the noncatalytic B subunit does not bind nucleotide and V_1_-ATPase from *Thermus thermophilus* HB8 showed strong MgADP inhibition and no steady-state activity [14]. Inspired by these observations, we hypothesized that strong MgADP inhibition of BF_1_ is due to the inability of noncatalytic sites to bind nucleotide. To examine this hypothesis, we prepared a mutant α_3_β_3_γ complex of BF_1_ in which nucleotide binding to the noncatalytic nucleotide binding sites can be monitored by the changes in the fluorescence from the tryptophan residues introduced near the noncatalytic sites. The result indicated that the noncatalytic sites of BF_1_ could bind ATP. Thus, the cause of strong MgADP inhibition of BF_1_ is not the weak binding ability of the noncatalytic sites but other steps required for the recovery from the MgADP inhibition. However, the mutant α_3_β_3_γ complex of BF_1_ that cannot bind nucleotide to the noncatalytic sites showed lowered ATPase activity, indicating that the nucleotide binding to the noncatalytic sites has a substantial role for recovery from MgADP inhibition in BF_1_.

## Materials and Methods

### Plasmid construction and protein preparation

The mutation (αR354W), which corresponded to the same mutant of *Escherichia coli* F_1_-ATPase (EF_1_) [15], was introduced by overlap extension PCR method [16] with KOD-plus DNA polymerase (Toyobo) and following primers by using the expression plasmid for the wild type (WT) α_3_β_3_γ complex of BF_1_, pET21-BF1 [12] as a template. Mutagenic primers were 5′-CTCAGGCGTATGGCCAGCGATCAATGCCGG-3′ and 5′-TTGATCGCTGGCCATACGCCTGAGAAGAAC-3′ and the franking primers were 5′-GCCGTATTGTAAACCCGCTAGGCCAG-3′ and 5′-TCTTGTGTGATGGCTGCTTGGCGAG-3′. The resulting 2.2 kbp DNA fragment was introduced into the *Eco*RV site of pZero2.1 vector (Novagen). Then the 0.8 kbp DNA fragment containing mutation was excised out by cutting this plasmid with *Not*I and *Nco*I. The fragment was put back to the original site of WT pET21-BF1 by ligating with *Nco*I/*Bam*HI fragment (1.2 kbp) and *Not*I/*Bam*HI fragment (7.3 kbp) of WT pET21-BF1. The resulting plasmid, pET21-BF1(αR354W) was used for protein expression. The mutations (αK175A/T176A), which is known to suppress nucleotide binding to the noncatalytic site [13], [17], were introduced in addition to αR354W by overlap extension PCR method with following primers by using pET21-BF1(αR354W) as a template. Mutagenic primers were 5′-CCGTCAAACAGGTGCAGCATCTGTTG-3′ and 5′- ATCGCAACAGATGCTGCACCTGTTTG-3′ and the franking primers were 5′-GAAATTAATACGACTCACTATAGG-3′ and 5′- GATAAGCACTCCGTAAAACCGAACTG-3′. The resulting 2.0 kbp DNA fragment was introduced into the *Eco*RV site of pZero2.1 vector (Novagen). Then the 1.6 kbp DNA fragment containing mutations was excised out by cutting this plasmid with *Xba*I and *Nco*I. The fragment was put back to the original site of pET21-BF1(αR354W) by ligating with *Nco*I/*Bam*HI fragment (1.3 kbp) and *Xba*I/*Bam*HI fragment (6.4 kbp) of pET21-BF1(αR354W). The resulting plasmid, pET21-BF1(αK175A/T176A/R354W) was used for protein expression. Mutations were confirmed by DNA sequencing. WT (α_3_β_3_γ^WT^), αR354W mutant (α_3_β_3_γ^RW^), and αK175A/T176A/R354W mutant (α_3_β_3_γ^ΔNC^) α_3_β_3_γ complexes of BF_1_ were prepared as described previously [12].

### Fluorescence measurement

The assay mixture consisted of 50 mM Tris-H_2_SO_4_ (pH 7.5), 50 mM K_2_SO_4_ and 2 mM MgSO_4_ was transferred to a quartz cuvette (1.5 ml). The cuvette was placed in a fluorescence spectrophotometer, FP-6500 (JASCO, Tokyo) and the temperature was controlled to 25°C. The α_3_β_3_γ complex of BF_1_ was added to 100 nM. The concentrated ATP-Mg solution (a mixture of equal molar ATP and MgSO_4_) was injected into the cuvette at the time indicated and the changes in the fluorescence were measured every 0.5 s or 1 s until the fluorescence reached a plateau. Excitation and emission wavelengths were set at 300 nm and 350 nm, respectively. Excitation and emission slit widths were 5 and 10 nm, respectively. The solution was stirred continuously during the measurement. Emission spectra were measured before and after the time-course measurement at a rate 50 nm/min.

### Fluorescence data analysis

The time course of the fluorescence was corrected for baseline with buffer. The fluorescence change at a plateau was plotted against the ATP concentration and fitted with the simple binding equation or the Hill equation by the computer software (Origin 9.0 J, Microcal Co.). The sum of two simple binding equations did not improve fitting (data not shown).

### ATPase assay

ATPase activity was measured by NADH-coupled ATP-regenerating system at 25°C as described previously [12]. Reaction rates were determined at 3–5 s (initial) and 12–13 min (steady state) after the start of the reaction. The reaction rate in the presence of 0.1% LDAO was determined 100–150 s after the addition of LDAO.

### Other methods

Protein concentration was determined by the method of Bradford [18] using bovine serum albumin as a standard. Chemicals were of the highest grades available.

## Results and Discussion

### Tryptophan fluorescence of α_3_β_3_γ^RW^ was completely quenched by the addition of ATP

**Figure 1:**
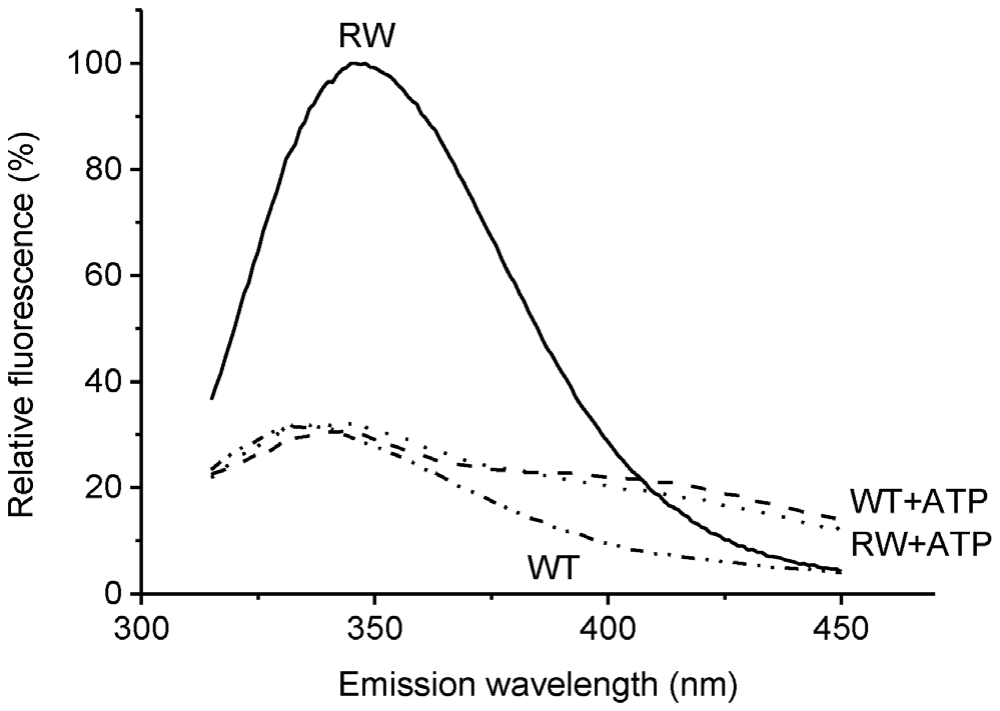
Emission spectra of α_3_β_3_γ^WT^ and α_3_β_3_γ^RW^ complexes of BF_1_. Fluorescence emission spectra of α_3_β_3_γ^WT^ and α_3_β_3_γ^RW^ complexes in the absence and presence of 1 mM ATP are shown. Excitation wavelength was 300 nm and the fluorescence emission spectra were recorded at 50 nm/min. Excitation and emission slit-widths were set at 5 and 10 nm, respectively. The fluorescence values are normalized to peak of α_3_β_3_γ^RW^ in the absence of ATP as 100%. Solid line, dotted line, two-dot-chain line and dashed line represent α_3_β_3_γ^RW^-ATP, α_3_β_3_γ^RW^+ATP, α_3_β_3_γ^WT^-ATP and α_3_β_3_γ^WT^+ATP, respectively.

**Figure 2:**
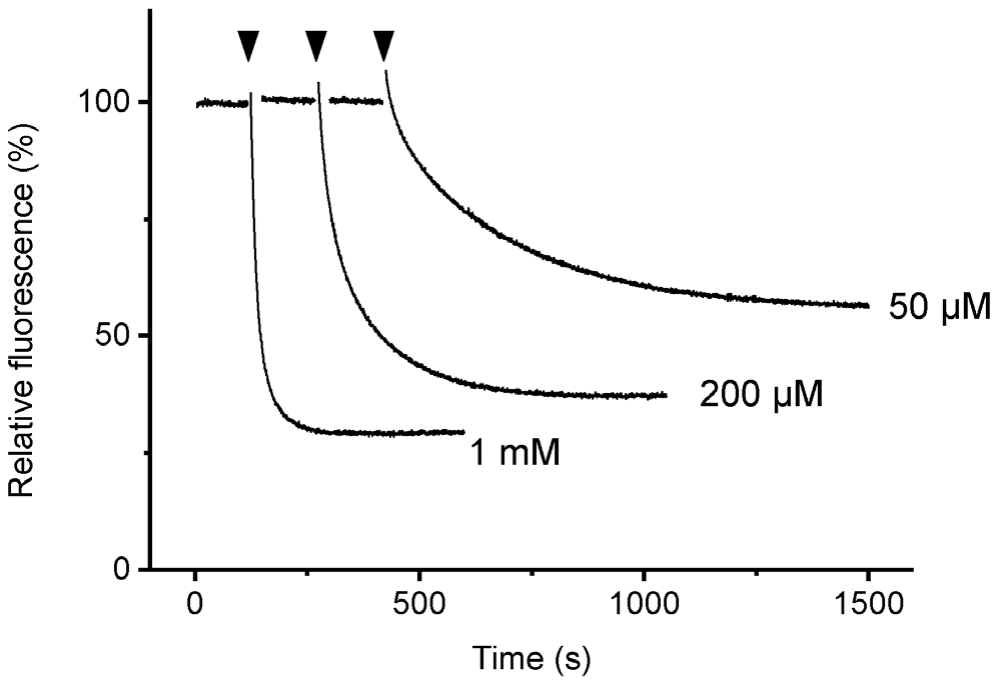
Time course of fluorescence change of α_3_β_3_γ^RW^ upon addition of ATP. MgATP was added at the times indicated by the arrowheads. Final ATP concentration in each measurement is shown on the right. Fluorescence is normalized to that before the addition of ATP.

**Figure 3:**
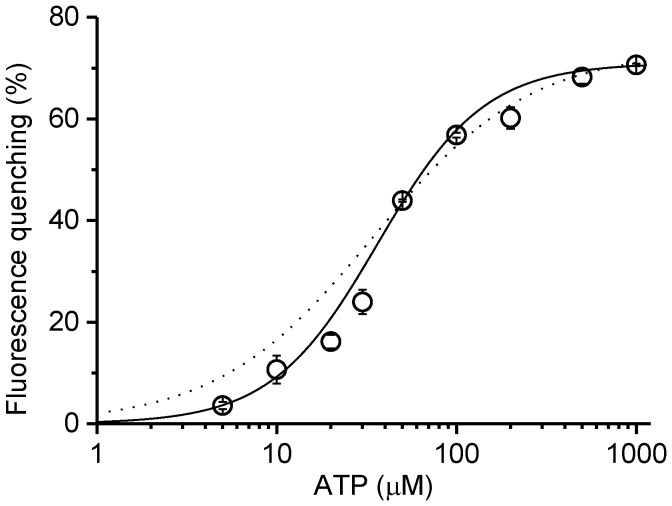
Titration of fluorescence by ATP. Changes in the fluorescence upon addition of ATP are expressed as percent of the fluorescence before the addition of ATP. Values are taken when the fluorescence reached a plateau. Error bars represent standard errors. The solid line represents the theoretical curve with the Hill equation (Fluorescence quenching  = *ΔFL*_max_×[ATP]^n^/(*K_d_* ^n^+[ATP]^n^)) with the following parameters(± standard error); *K_d_* = 36.5±1.2 µM, *ΔFL*_max_ = 71.0±0.7%, *n* = 1.47±0.1. The dotted line represents the theoretical curve with simple binding equation (Fluorescence quenching  = *ΔFL*_max_×[ATP]/(*K_d_*+[ATP])) with *K*_d_ = 34.4±2.9 µM, *ΔFL*_max_ = 73.5±1.5%.

**Figure 4:**
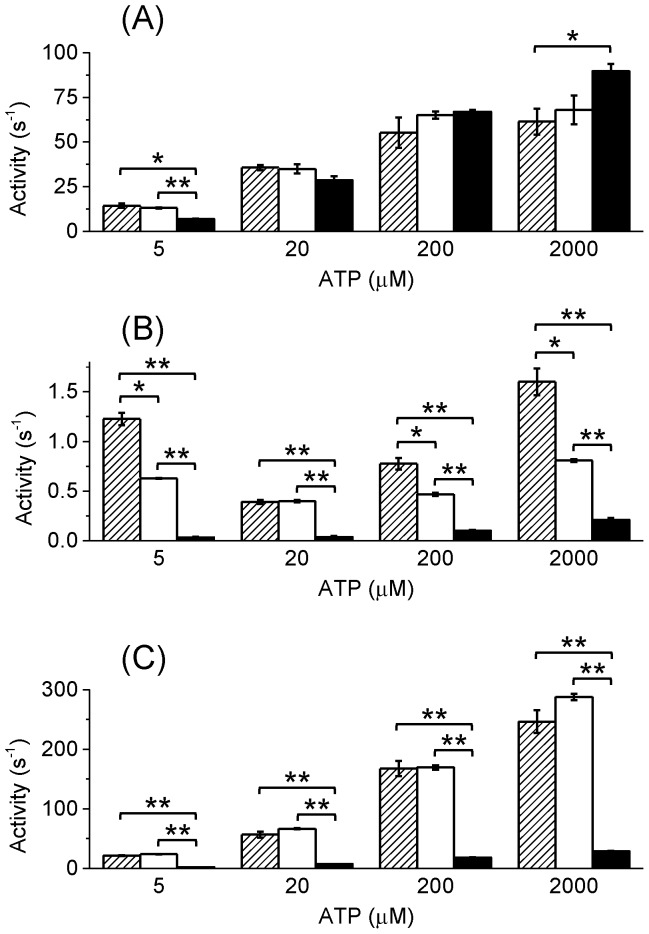
ATPase activities of α_3_β_3_γ complexes. The initial (A) and steady-state (B) ATPase activities and ATPase activity in the presence of 0.1% LDAO (C) were determined. Hatched, open and solid bars represent α_3_β_3_γ^WT^, α_3_β_3_γ^RW^ and α_3_β_3_γ^ΔNC^, respectively. Error bars represent standard errors. Asterisks represent statistically significant differences (*p<0.05 and **p<0.01, Student's t-test). Data for WT are taken from [12].

The mutant α_3_β_3_γ^RW^ showed large fluorescence compared to the WT (Fig. 1). Addition of ATP resulted in the quenching of fluorescence to the same level as the WT background, indicating that the fluorescence from tryptophan introduced near the noncatalytic sites was completely quenched by the addition of ATP. Thus, as reported on EF_1_ [15], tryptophan fluorescence could be used as the indicator of nucleotide binding to the noncatalytic sites of the α_3_β_3_γ^RW^ complex. The time course of fluorescence showed the ATP-concentration dependent rate and magnitude of fluorescence quenching (Fig. 2). There was a small jump in the fluorescence upon addition of ATP but this was not considered in the calculation of the degree of quenching. The titration with ATP showed an apparent *K*_d_ = 34.4 µM with simple binding equation and an apparent *K*_d_ = 36.5 µM and *n* = 1.47 with Hill equation (Fig. 3). These values were in the same range as that reported on EF_1_ (*K*_d_ of the noncatalytic site for MgATP is 25 µM) [15]. It should be noted that the part of ATP will be hydrolyzed into ADP and Pi and the noncatalytic sites will be filled with some combination of ATP and ADP depending on initial ATP concentration since the fluorescence measurement did not include pyruvate kinase, an ATP-regenerating enzyme. Nevertheless, according to the results of ATPase measurement (Fig. 4), only a few percent of ATP was hydrolyzed before fluorescence reached the plateau (∼100 s) at high concentrations of ATP such as 1 mM.

### α_3_β_3_γ^RW^ Was inhibited severely even the noncatalytic sites were filled

Except for the lower steady-state activity, the mutant α_3_β_3_γ^RW^ showed similar ATPase properties to the α_3_β_3_γ^WT^ (Fig. 4); very high initial activity and strong inactivation to approximately 1% of the initial activity, and activation by LDAO more than 300-fold at 2 mM ATP for example. The lower steady-state activity may be due to the altered affinity or specificity of noncatalytic site by the mutation, although the fluorescence measurements (Fig. 3) indicate that ATP should fill noncatalytic sites in the steady-state (12–13 min after the start of the reaction) ATPase measurement at >200 µM ATP. Thus, BF_1_ was strongly inhibited by the MgADP inhibition even when the noncatalytic sites were filled with ATP. Although ATP and ADP could affect differently on releasing MgADP inhibition as reported on chloroplast F_1_ [19], [20], noncatalytic sites of BF_1_ must be filled with ATP during the ATPase measurement because our ATPase measurement contained ATP-regenerating system.

### α_3_β_3_γ^ΔNC^ Showed even lower ATPase activity

Since there are no obvious correlation between rate of very rapid inactivation [12] and that of nucleotide binding to the noncatalytic sites (Fig. 2), it was unclear that whether the nucleotide binding to the noncatalytic sites could facilitate release of inhibitory MgADP or not. To clarify this, we prepared the mutant α_3_β_3_γ complex of BF_1_ that contained mutations in Walker A motif (α_3_β_3_γ^ΔNC^) to test if the nucleotide binding to the noncatalytic sites of BF_1_ promotes recovery from MgADP inhibition, even if weak. With the α_3_β_3_γ^ΔNC^, no fluorescence quenching upon addition of ATP was observed (Fig. S1), indicating that the mutation totally abolished nucleotide binding to the noncatalytic sites as reported on TF_1_ [13]. The initial ATPase activity of α_3_β_3_γ^ΔNC^ complex was essentially the same level as the WT (Fig. 4A). However, the steady-state ATPase activity was much lower (2.7∼13% of WT, Fig. 4B). Even in the presence of LDAO, the activity was very low compared to the WT (10∼13% of WT, Fig. 4C). These properties of the α_3_β_3_γ^ΔNC^ complex were similar to those reported on the same mutant of TF_1_ [13], [21], suggesting that the noncatalytic site of BF_1_ also has the substantial role to facilitate the release of inhibitory MgADP from the catalytic sites even if low efficiency.

## Conclusions

The noncatalytic nucleotide binding sites of BF_1_ can bind nucleotides by the affinity similar to other F_1_-ATPases. From the result, there rose the possibility that the nucleotide binding to noncatalytic site of BF_1_ does not affect release of MgADP inhibition. However, this was not the case because the α_3_β_3_γ^ΔNC^ mutant had even lower steady-state activity than the WT or the RW mutant. Thus, the most part of strong MgADP inhibition of BF_1_ is due to the different factors. Inefficient transmission of the conformational changes from the noncatalytic sites to the catalytic sites induced by the binding of ATP to the noncatalytic sites [22] or the intrinsic propensity of catalytic β subunit, for example, are the candidates. Following results presented here, we are carrying out the experiments that may determine the part responsible for the strong MgADP inhibition of BF_1_ from the different point of view. Although the MgADP inhibition is common to all ATP synthases, the degree of that varied considerably. There have been no report about such strong MgADP inhibition on other F_1_-ATPases. To study what determines the degree of the MgADP inhibition may help us to understand the whole picture of the physiological regulation of ATP synthases from various species living in the various environment.

## Supporting Information

Figure S1 **Emission spectra of α_3_β_3_γ^ΔNC^ of BF_1_.** Fluorescence emission spectra of α_3_β_3_γ^ΔNC^ in the absence and presence of 1 mM ATP were measured as Fig. 1. Solid line and dotted line represent in the absence and presence of ATP, respectively. The fluorescence values are normalized to peak in the absence of ATP as 100%.
(TIF)
